# Early Ocular Response to Visual Motion in Patients With Persistent Postural‐Perceptual Dizziness

**DOI:** 10.1111/nyas.70378

**Published:** 2026-08-12

**Authors:** Vergil V. Mavrodiev, Konrad P. Weber, Christopher J. Bockisch, Fabienne C. Fierz

**Affiliations:** ^1^ Department of Neurology University Hospital Zurich Zurich Switzerland; ^2^ Department of Ophthalmology University Hospital Zurich Zurich Switzerland; ^3^ Department of Otorhinolaryngology University Hospital Zurich Zurich Switzerland

**Keywords:** dizziness, PPPD, vertigo, vestibular, VID

## Abstract

Persistent postural‐perceptual dizziness (PPPD) patients with visual dependency rely more on visual cues and may have altered oculomotor responses to visual motion. This study examined early eye movement responses to large‐field motion stimuli—the ocular following responses (OFRs)—in 30 PPPD patients and 29 healthy controls. Participants viewed horizontal motion in peripheral and/or central visual fields while eye movements were recorded. OFR velocity 200 ms after stimulus onset and suppression efficacy were analyzed across four central field sizes from 5° to 42° radius. Both groups exhibited strong OFRs to stimuli in the central visual field that increased with the size of the stimulation area. When the central visual field was stationary and the surrounding area moved, patients showed significantly higher eye velocity than controls. Both groups could partially suppress OFRs, but healthy controls were more effective at suppressing eye movements during peripheral stimulation. Patients also showed greater visual dependency on the dynamic subjective visual vertical (2.2° vs. 1.2°), which correlated with OFR magnitude. Overall, PPPD patients were less able to keep their eyes still during peripheral visual motion, producing greater retinal slip and disrupting volitional fixation—an abnormality that may contribute to dizziness and discomfort in visually complex environments.

## Graphical abstract

When the central visual field was held stationary and the surround moved, PPPD patients with visual dependency showed significantly larger ocular following responses (OFRs) than healthy controls and were less able to suppress them, as measured 200 ms after motion onset. This impaired suppression of reflexive following to peripheral motion produces greater retinal slip and disrupts volitional fixation. These findings point to altered early visual motion processing—and difficulty suppressing visual reflexes to peripheral motion—as a mechanism underlying visually induced dizziness in complex environments.

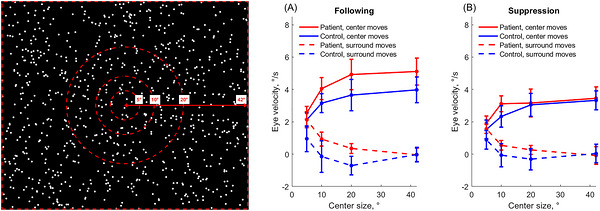

## 1 Introduction

The human visual system plays a critical role in spatial orientation through its capacity to detect and interpret motion signals across the visual field. The ocular following response (OFR) is a reflexive eye movement triggered by sudden large‐field motion [1, 2, 3, 4, 5]. It has been postulated that the OFR represents one of the earliest components of the optokinetic reflex while still differing from smooth pursuit, the latter one being more strongly affected by attentive and cognitive cues [6, 7, 8]. The OFR originates in direction‐selective neurons of the medial superior temporal (MST) cortex, which project via the dorsal pontine nuclei to brainstem ocular motor nuclei [2]. This 70–85 ms reflex arc precedes conscious motion perception, providing a window into preattentive visual processing [9, 10].

Visual dependency, a maladaptive overreliance on visual cues, manifests clinically as visually induced dizziness and is a hallmark of persistent postural‐perceptual dizziness (PPPD) [11, 12]. Patients report debilitating symptoms in environments with complex visual motion (e.g., crowded spaces, scrolling screens, supermarket aisles), yet standard vestibular testing often reveals intact peripheral function [13]. This paradox suggests an altered function in higher‐order sensory integration rather than primary sensory dysfunction. Supportive of this view is the finding that PPPD patients show exaggerated postural sway to full‐field motion [14, 15, 16, 17, 18]. While gaze stability following prolonged viewing of moving patterns decreases in PPPD patients [19], other types of oculomotor behavior in PPPD remain unexplored, despite the fact that atypical ocular responses to visual stimuli could produce visual motion signals that cause or exacerbate the symptoms of visual dependency. Such responses could be symptomatic through two complementary routes: by increasing retinal slip and blurring vision, and by interfering with the volitional task of maintaining fixation, so that difficulty suppressing reflexive responses to visual motion in motion‐rich environments is itself a source of symptoms.

Recent theories propose that PPPD symptoms arise from mismatches between sensory predictions and actual input [20]. The OFR, being a preattentive reflex, provides a window into early visual motion processing prior to cognitive modulation [3, 21]. In healthy individuals, OFR magnitude depends on stimulus characteristics [22, 23, 24] (e.g., field size, contrast) and can be partially suppressed volitionally through fixation [25]. We hypothesize that visual dependency reflects both enhanced gain in early motion processing pathways and impaired top‐down control of reflexive visuomotor responses.

This study investigates two key questions:

1. Do patients with PPPD and visual dependency exhibit amplified OFRs compared to controls, and does the response change depending on whether the central or peripheral visual field is stimulated?
2. Is the ability to suppress OFRs through conscious fixation diminished in these patients?

## 2 Materials and Methods

The ethics committee of the canton of Zurich approved the study protocol (BASEC‐Nr.2022‐01399) in adherence to the Declaration of Helsinki, and all participants provided written informed consent.

### 2.1 Participants

Patients were recruited from the Interdisciplinary Center for Vertigo and Neurological Visual Disorders at the University Hospital Zurich. All patients met the Bárány Society diagnostic criteria for PPPD with prominent visually induced symptoms, as confirmed by clinical assessment and an abnormal dynamic subjective visual vertical (SVV) test [12, 26]. All participants were at least 18 years of age, provided informed consent, had normal or corrected‐to‐normal vision, and no history of visual‐oculomotor disorders. Controls had no history of vestibular disorders. Psychiatric history was not an exclusion criterion for either group.

### 2.2 Procedures

Participants sat 1 m in front of a 179 × 143 cm screen, subtending 84° × 71° of visual angle. Eye movements were recorded monocularly at 220 Hz with a head‐mounted video‐oculography device (EyeSeeCam v2011, EyeSeeTec, Munich, Germany). Visual stimuli were generated in MATLAB (version 2015, Natick, MA: The MathWorks Inc.) with Psychophysics Toolbox extensions [27, 28]. The eye tracker was calibrated by having participants fixate on five calibration targets (0, ± 9° horizontally and vertically).

**FIGURE 1:**
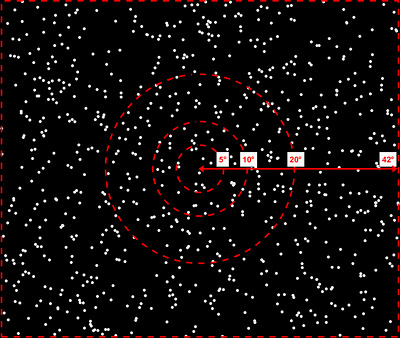
Participants viewed a display with randomly placed dots. The display was separated into different‐sized center and surround regions (boundaries shown in red), and in different trials, the dots in either the center or the surround moved horizontally at ± 25°/s. Trials in which all the dots (42°) either moved or were stationary were also included.

The motion stimuli consisted of a dense field of randomly positioned white dots (approximately 800 dots, 6 pixels wide, ∼0.35°; 0.13 dots per square degree) on a dark background (Figure 1). All dots were initially stationary, and after a random delay of 1–2 s, a portion of the dots moved left or right at 25°/s for 0.5 s. The screen was divided into a central circular region, which varied in size from 5°, 10°, and 20° in radius, and a surrounding region. Dots in either the central or surrounding region would move. Additionally, trials where either all dots moved (considered to have a 42° radius based on the horizontal screen size) or were stationary were included. Trials in which the central dots moved while the surround stayed still are referred to as “center moves”; trials in which the surround moved while the central region stayed still are referred to as “center stationary.”

Two experimental conditions were tested: “following” and “suppression.” To minimize ambiguity, when referring to the ocular “following” and ocular “suppression” trials in this paper, the words are put in quotes. As the OFR is more pronounced shortly after saccades, “following” trials began with the appearance of a red fixation cross (1.5 deg diameter, with lines 0.5° wide), 10 degrees either to the right or left of center (chosen randomly), and after a random delay between 1 and 2 s, jumped to the center [3]. The central fixation cross disappeared after 300 ms, and the central or surrounding dots moved for 0.5 s. The dots then vanished, and the fixation cross reappeared for the next trial. Participants were instructed to look at the red cross when visible and to allow their eyes to follow the moving dots when the cross disappeared.

In “suppression” trials, which were always completed after “following” trials, a green fixation cross (1.5 deg in diameter, with lines 0.5° wide) flashed in the center of the screen for 1/60 of a second every 3–4 s. Participants were instructed to keep looking at the center of the screen and to actively suppress any following eye movements when the dots moved. The motion stimulus was identical to that in the ocular “following” trials.

Ocular “following” trials were conducted in two blocks of 80 trials, resulting in 10 trials for each condition (4 center sizes × 2 center/surround movement × 2 directions of movement). “Suppression” trials were similarly structured. One patient completed only one block of suppression trials due to discomfort.

All participants also completed a dynamic SVV test to measure visual dependency [26]. Participants viewed a computer screen from 30 cm through a circular tube that blocked visible references such as the edges of the computer monitor. Throughout each trial, a field of white dots (1° to 3.4° in diameter) rotated continuously at 60°/s, providing a moving visual background. While the dots were rotating, an arrow (36° long) appeared at the center of the screen at a random orientation, and participants used key presses to rotate it until it appeared vertical. Six trials with clockwise background rotation and six with counterclockwise rotation were presented in a random order. We expected that the rotating dots would cause subjects to misperceive the orientation of the line in the direction of the dot motion, so they would adjust the line rotation into the opposite direction to compensate. We calculated the mean SVV for each rotation direction and then used the difference of the means (counterclockwise—clockwise) divided by two as a measure of visual dependency.

Participants further completed the visual vertigo analogue scale (VVAS) to record their symptoms prior to testing [29]. Participants rated nine common dizziness‐provoking activities (e.g., being a passenger in a car or watching a movie) on a scale from 0 (no dizziness) to 10 (most severe dizziness). We computed the average score on all answered items for each participant.

### 2.3 Data Analysis

Data were analyzed with MATLAB v2022b (The MathWorks Inc.). Raw eye position data were smoothed with a first‐order Savitzky−Golay filter (span = 0.0227 s). Saccades were identified using a velocity threshold of 25°/s. The beginning of each saccade was marked at two data points (∼0.01 s) before eye velocity exceeded 5°/s, and the end was marked five samples (∼0.0227 s) after eye velocity dropped below 5°/s.

Trials where a blink or saccade occurred during the first 200 ms after motion onset were discarded. Our purpose was to measure the early OFR with as little influence of visual‐feedback mechanisms as possible. Even smooth‐pursuit reaction times, which are generally longer than those of the OFR, have been measured at 150–200 ms [30], and we anticipated values at the short end of this range because following is enhanced shortly after saccades (as indeed occurred; see Figure 4) [31]. Because our reaction times were generally in the 150–170 ms range, extending the analysis window to 250 ms would have increased the contribution of visual‐feedback mechanisms. Overall, 12% of trials from healthy subjects and 17% of trials in patients contained either a saccade or a blink in the first 200 ms after motion onset and were not analyzed.

Data for leftward motion were mirrored and combined with data for rightward motion. Reaction time was defined as the time when eye velocity exceeded 1°/s and then remained above 1°/s for 100 ms. The early eye movement response was defined as the eye velocity 200 ms after pattern movement onset, and for each participant, we averaged the eye velocity for each stimulus type.

Repeated measures analysis of variance was performed using R Statistical Software (v4.4.1; R Core Team 2024) with Greenhouse−Geisser sphericity correction. Multiple comparison tests were conducted using Holm's method [32]. Tests for normal distributions were made with the Anderson−Darling test, and either *t*‐tests and Pearson correlations, or Wilcoxon rank‐sum and Spearman correlations were calculated in MATLAB [33]. To account for the age difference between groups, all repeated measures analyses were additionally performed with age included as a covariate.

## 3 Results

Thirty patients with PPPD and visual dependency and 29 healthy controls participated. One participant from each group was excluded due to excessive blinking, which interfered with video eye tracking. The patient group had a mean age of 55 years (range 34–69, *SD* = 11 years) and included 19 females. The control group had a mean age of 33 years (range 23–72, *SD* = 13 years) and included 18 females.

Patients scored significantly higher (Wilcoxon rank‐sum = 448, *p* < 0.001) on the VVAS, with a mean of 4.5 out of 10 (*SD* = 2.2), while controls had a mean of 0.3 (*SD* = 0.4).

**FIGURE 2:**
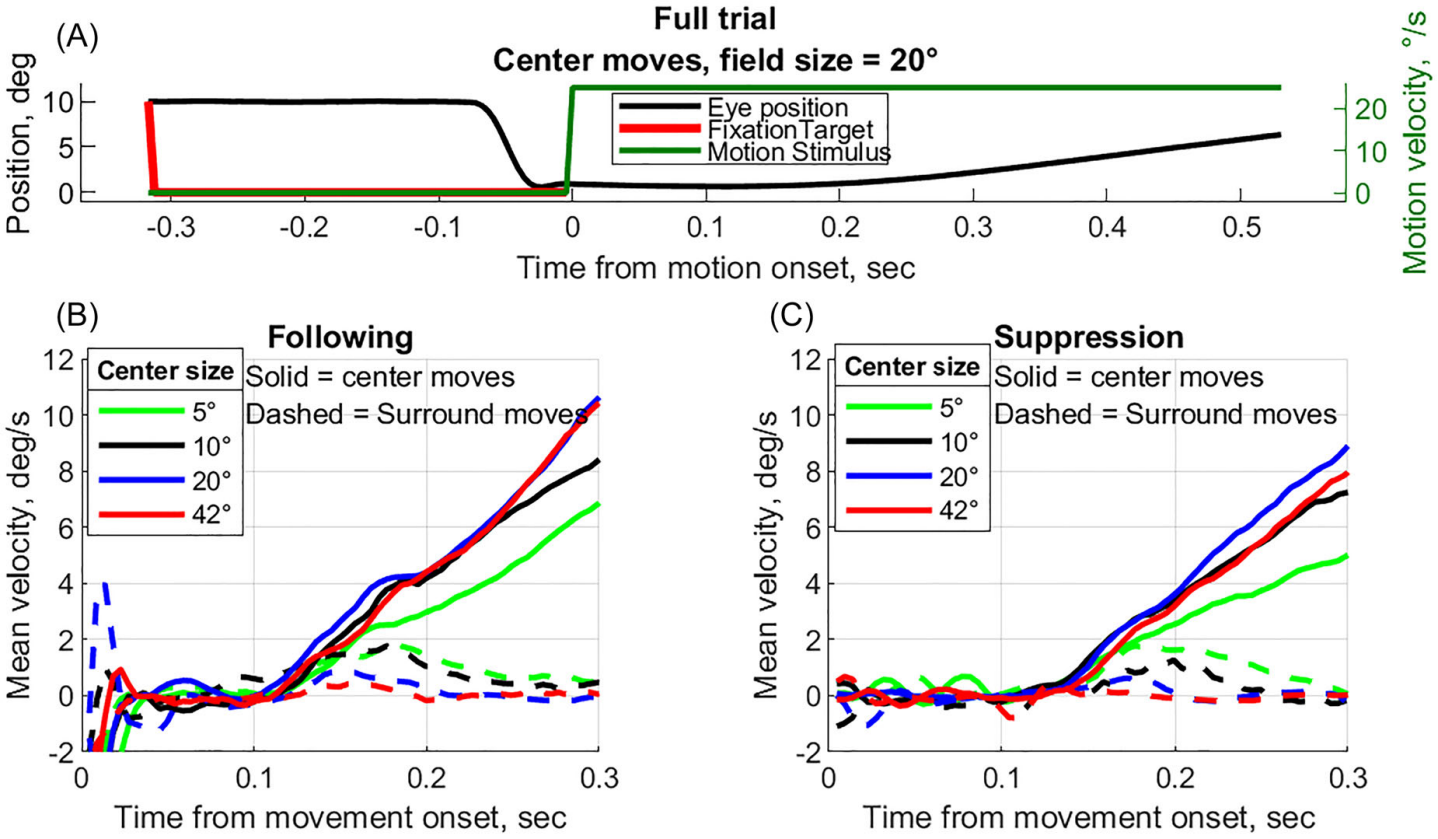
(A) Example single‐trial patient data showing the fixation target (red) stepping from 10° to 0° at −0.3 s, the participant making a saccade (black) to refixate the target at about –0.1 s, the dots within a 20° radius of center start to move at time = 0 s at 25°/s. Average eye velocity, for the same patient, in the “following” (B) and “suppression” conditions (C).

Example data from a patient is shown in Figure 2. Panel A displays a representative trial where the participant makes a leftward saccade just before the dots in the central 20° region move. After a delay of ∼150 ms, the eye begins to move smoothly in the direction of dot movement. Panel B summarizes all the “following” trials for this patient. Eye velocity began to increase ∼125 ms after dot motion onset. When the center dots moved, eye velocity increased throughout the trial, whereas when surrounding dots moved, eye velocity initially increased and then returned toward zero. Panel C shows results from “suppression” trials, which were largely similar except that eye velocity was slightly lower at the end of the trials.

**FIGURE 3:**
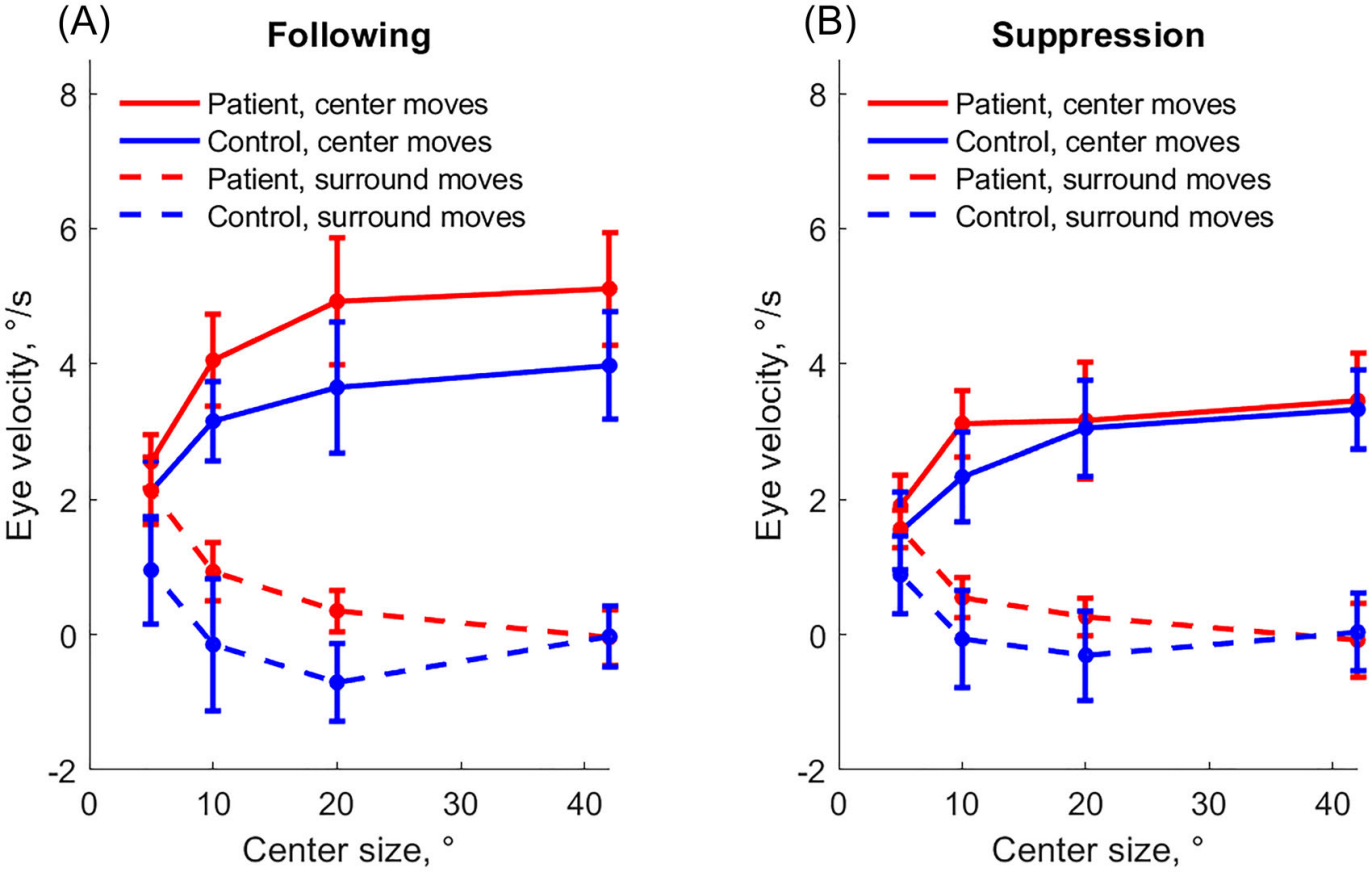
Eye velocity 200 ms after the pattern movement started, averaged across participants. (A) “Following” trials. (B) “Suppression” trials. Error bars are 95% confidence intervals.

We quantified the early eye movement response as the velocity 200 ms after pattern movement began. Given that reaction times averaged approximately 150 ms, this measurement represents eye velocity approximately 50 ms after the start of smooth eye movement. Figure 3 shows the average response across all participants. In the “following” condition (Figure 3A), eye velocity increased with region size, reaching peak velocity of ∼5°/s for patients and ∼4°/s for controls, and appears to saturate for larger field sizes. When the surround moved, eye velocity was highest when the central region was small and approached 0°/s when the entire screen was stationary. In both conditions, patients had higher eye velocity than controls.

We performed a repeated measures analysis of variance with group (patient and control), field size, center/surround movement as factors and age as a covariate on the “following” data. Main effects of group (*F* = 4.4, *df* = 1.56, *p* = 0.039), field size (*F* = 4.85, *df* = 3.168, *p* = 0.003), and center/surround movement (*F* = 16.3, *df* = 1.56, *p* < 0.001) were significant, while the main effect of age was not (*F* = 0.22, *df* = 1.56, *p* = 0.63). The interaction effects of age and field size (*F* = 3.9, *df* = 3.168, *p* = 0.009) and field size and center/surround movement (*F* = 3.89, *df* = 2.35, 131, *p* = 0.018) were significant, while all other interactions were not significant. ANOVAs conducted separately on the “center moves” and “center stationary” conditions found that the effect of group was significant when the center was stationary (*F* = 7.64, *df* = 1.56, *p* = 0.008), but the effect of group did not reach significance when the center moved (*F* = 1.81, *df* = 1.56, *p* = 0.184). The main effect of size was significant when the center moved (*F* = 6.17, *df* = 2.42, 135, *p* = 0.001) but not when the center was stationary (*F* = 2.17, *df* = 2.58, 144.4, *p* = 0.174).

When the surround moved, both healthy participants and patients exhibited eye velocity significantly greater than zero when the central region was small (5°) (*p*‐values < 0.001, Holm's corrected). While healthy participants did not show a response for all larger regions (all *p*‐values > 0.7, Holm's corrected), patients showed eye velocity greater than zero (all *p*‐values > 0.02, Holm's corrected) for all field sizes except for when the entire visual field was stationary.

Figure 3B summarizes the “suppression” data. The repeated measures ANOVA found significant main effects for field size (*F* = 4.88, *df* = 2.35, *p* = 0.006), and center/surround movement (*F* = 9.3, *df* = 1.56, *p* = 0.004) but not for group (*F* = 0.03, *df* = 1.56, *p* = 0.86) or age (*F* = 3.67, *df* = 1.56, *p* = 0.061). There were significant interactions for group and size (*F* = 4.3, *df* = 2.35, 131.7, *p* = 0.004) and age and size (*F* = 5.2, *df* = 2.35, 131.7, *p* = 0.011), and all other interaction effects were not significant. The interaction of group and size appears to occur because the difference between groups is smaller for the largest field sizes (and perhaps smallest) field size.

When the central region was small and stationary, the eye velocity of both healthy participants and patients was greater than zero (*t*‐tests, Holm's corrected *p*‐values < 0.05). With larger field sizes, the eye velocity of healthy participants was never significantly greater than zero (all Holm's corrected *p*‐values > 0.6), whereas in patients, eye velocity when the central region was 10° was also significantly greater than zero (*t*‐tests, Holm's corrected *p*<0.001) and approached significance when the central region was 20° (*p* = 0.065).

To determine if attempted suppression was in fact successful in reducing eye velocity, for each participant, we computed the change in velocity, and for each field size, we conducted *t*‐tests (corrected for multiple comparisons with Holm's correction) for if the reduction in velocity was greater than zero. In all cases when there was central motion, except for one (healthy subjects with a 20° field size), the reduction in eye velocity was significantly greater than zero (all *p*s < 0.038, Holm's corrected). (When the surround moved, there was only one case where the reduction in velocity was significant, patients with a 10° field size, *p* = 0.002, and one case that approached significance, patients with a 20° field size, *p* = 0.06.) We next conducted a repeated measures ANOVA on the center movement data with group, center/surround motion, field size, and age as factors. The main effects of group (*F* = 5.1, *df* = 1.56, *p* = 0.028) and field size (*F* = 7.6, *df* = 2.36, 132.1, *p* <0.001) were significant, while the effect of center/surround was marginal (*F* = 3.7, *df* = 1.56, *p* = 0.06) and the effect of age was not significant. The group by field size interaction (*F* = 5.64, *df* = 2.36, 132.1, *p* = 0.003) and age by field size interaction (*F* = 8.2, *df* = 2.36, 132.1, *p*<0.001) were significant, and all other interaction effects were nonsignificant.

**FIGURE 4:**
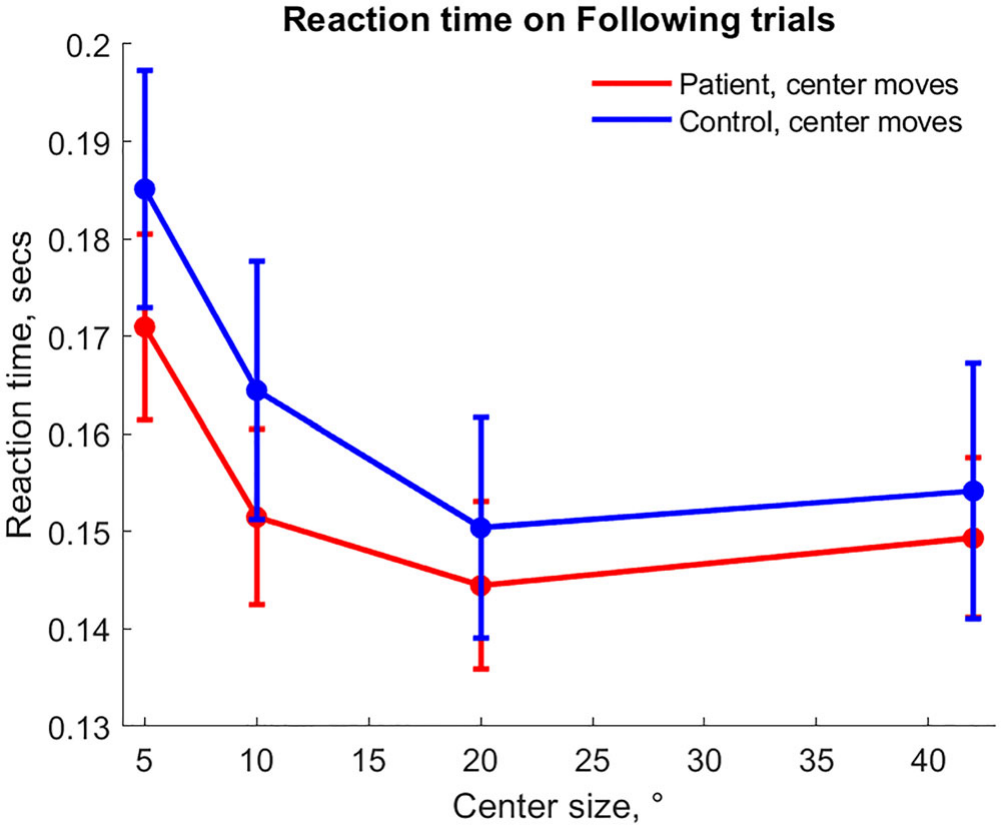
Reaction time on “following” trials when there was motion in the central visual field, averaged across participants. Error bars are 95% confidence intervals.

We analyzed reaction times only for trials where the central dots moved (Figure 4), as eye velocity often did not meet our onset criteria (1°/s for 100 ms) when only the surrounding dots moved. Reaction times did not differ significantly between groups (healthy mean = 0.164 s, *SD* = 0.03; patient mean = 0.154 s, *SD* = 0.03) (ANOVA, *F* = 2.1, *p* = 0.152, *df* = 1.56).

The average dynamic SVV was greater in patients (2.2°) than in controls (1.2°) (Wilcoxon rank‐sum = 1027, *p* = 0.009). For patients, there was a significant positive correlation between the average velocity of the OFR when the central dots moved (averaged across all field sizes) and dynamic SVV deviation (Spearman rho = 0.45, *p* = 0.03). This correlation was not significant in controls (Spearman rho = −0.002, *p* = 0.993).

## 4 Discussion

We investigated whether PPPD patients with visual dependency show altered OFRs compared to healthy controls. Our findings reveal several key differences in OFRs between these groups, supporting the hypothesis that enhanced early visual motion processing may contribute to the symptoms experienced by patients with PPPD.

Patients showed enhanced OFRs than controls when the surround moved while the central visual field was held stationary. This group difference remained significant after age was entered as a covariate, with age itself not a significant predictor. By contrast, the group difference for central motion, although visible in the raw velocities (Figure 3A), did not survive age adjustment and should be regarded as age‐sensitive. The most notable result, therefore, lay not in how strongly patients followed central motion, but in their reduced ability to keep the eyes still when the periphery moved. The qualitative pattern of responses across field sizes and for center versus surround motion was otherwise similar in both groups, suggesting that the basic organization of the OFR system is intact in patients with PPPD. The enhanced following to central motion is unlikely to be deleterious to foveal image stability, since in both groups eye velocity remained below stimulus velocity, so the response aids rather than disrupts tracking. Whether steady‐state following gain approaches unity and visual acuity is preserved during sustained motion could be tested in future work.

Both healthy controls and patients could partially suppress OFRs. When the central visual field was stationary, healthy controls were able to suppress OFRs such that eye velocity was not significantly different from zero except for the smallest central visual field sizes. Patients, on the other hand, continued to show significant eye velocity for moderate‐sized visual fields (10° and 20°). Beyond the retinal slip this produces, the impaired suppression of reflexive following points to a second, complementary mechanism: interference between the reflexive OFR and the conscious task of maintaining fixation. In motion‐rich environments, difficulty suppressing these visual reflexes may itself provoke visually induced dizziness—retinal slip blurs vision, while the disruption of volitional fixation may be the more fundamental problem. Suppressing such eye movements to peripheral motion is necessary to maintain clear vision when fixing on an object that moves with the observer, for example, when attempting to read a wristwatch or phone while walking, or when fixating a distant object while linearly translating.

Reaction times for the OFR did not differ significantly between both groups, suggesting that the timing of the initial visual motion detection remains normal in patients with PPPD, and the enhanced responses in patients likely reflect differences in oculomotor gain.

The dynamic SVV test results confirmed greater visual dependence in the patient group, with significantly larger deviations (2.2° vs. 1.2°) during background rotation. Moreover, we found a significant positive correlation between OFR magnitude and SVV deviation in patients but not in controls, suggesting that enhanced early visual motion processing may be directly connected to the broader phenomenon of visual dependency in these individuals.

Recent neuroimaging studies have identified alterations in brain connectivity in patients with chronic dizziness, particularly involving visual motion processing areas and regions associated with spatial orientation [34, 35, 36]. The enhanced OFRs observed in our study may reflect increased excitability or reduced inhibition within these neural circuits. More specifically, the OFR is generated by direction‐selective neurons of the MST that project through the dorsal pontine nuclei to the brainstem ocular motor system, so an enhanced response may reflect altered gain along this pathway. The difficulty in suppressing reflexive following, by contrast, may implicate the insular and parieto‐insular vestibular networks that support visual attention and have been linked to the symptoms of chronic dizziness [37].

In the clinic, the OFR may help to illuminate the physiology of visual dependency and could serve as a candidate measure for tracking the effectiveness of therapeutic interventions, like those done with vestibular migraine [38]. Current diagnostic approaches rely heavily on subjective symptom reports and questionnaires, and an objective physiological measure would be valuable. We caution, however, that the modest effect sizes observed here make it unlikely that the OFR would achieve the sensitivity and specificity required of a stand‐alone diagnostic test; its principal value lies in elucidating the physiological mechanisms of visually induced dizziness. In addition, the residual eye movements to peripheral motion could be targeted with rehabilitation strategies emphasizing fixation control with peripheral motion stimulation, like other successful strategies employing optokinetic stimulation [39].

## 5 Conclusion

Patients with PPPD and visually induced dizziness exhibit enhanced OFRs to visual motion, in particular when the peripheral visual field is stimulated, which could suggest increased difficulty in maintaining clear foveal vision during attempted fixation while moving. These findings suggest that visual dependency involves altered early visual motion processing, which may contribute to the symptoms experienced by these patients in visually complex environments. Future research should explore the neural mechanisms underlying these enhanced responses and investigate whether targeted rehabilitation strategies can normalize these altered visual motion responses.

## 6 Limitations

Several limitations of our study should be acknowledged. First, the patient and control groups were not age‐matched, with patients being, on average, older than controls. However, in adulthood, oculomotor gains are generally stable or decline with age [40, 41, 42, 43]. Second, our PPPD patient group was heterogeneous in terms of the precipitating conditions that led to their visual dependency, which could introduce variability in the underlying mechanisms. Future studies might benefit from more homogeneous patient populations and from stratifying analyses based on precipitating conditions. Finally, the conditions of the “following” and “suppression” conditions were not exactly matched, as participants performed a saccade task in the “following” condition but not in the “suppression” condition. Direct comparisons between the two should, therefore, be treated cautiously.

## Author Contributions

Conceptualization: C.J.B. and F.C.F. Data collection: V.V.M. and C.J.B. Methodology: V.V.M., K.P.W., C.J.B., and F.C.F. Formal analyses: V.V.M. and C.J.B. Writing – original draft: V.V.M. and C.J.B. Writing – review and editing: V.V.M., K.P.W., C.J.B., and F.C.F. Visualization: V.V.M. and C.J.B. Supervision: K.P.W. and F.C.F.

## Conflicts of Interest

The authors declare no conflicts of interest.

## Data Availability

The datasets used for the current study are available from the corresponding author upon reasonable request. The data availability may be subject to approval from the ethics committee.
